# Primary care physicians’ educational needs and learning preferences in end of life care: A focus group study in the UK

**DOI:** 10.1186/s12904-017-0191-2

**Published:** 2017-03-09

**Authors:** Lucy Ellen Selman, Lisa Jane Brighton, Vicky Robinson, Rob George, Shaheen A. Khan, Rachel Burman, Jonathan Koffman

**Affiliations:** 1 University of Bristol, School of Social and Community Medicine, University of Bristol, 39 Whatley Road, Bristol, BS8 2PS UK; 2 King’s College London, Cicely Saunders Institute, Bessemer Road, Denmark Hill, London, SE59PJ UK; 3 St Christopher’s Hospice, 51-59 Lawrie Park Road, London, SE26 6DZ UK; 4 Guy’s & St Thomas’ NHS Foundation Trust, Great Maze Pond, London, SE19RT UK; 5 King’s College Hospital NHS Foundation Trust, Bessemer Road, Denmark Hill, London, SE5 9RS UK

**Keywords:** General Practice, Primary Health Care, Education, End of Life Care, Palliative Care, Qualitative Research

## Abstract

**Background:**

Primary care physicians (General Practitioners (GPs)) play a pivotal role in providing end of life care (EoLC). However, many lack confidence in this area, and the quality of EoLC by GPs can be problematic. Evidence regarding educational needs, learning preferences and the acceptability of evaluation methods is needed to inform the development and testing of EoLC education. This study therefore aimed to explore GPs’ EoLC educational needs and preferences for learning and evaluation.

**Methods:**

A qualitative focus group study was conducted with qualified GPs and GP trainees in the UK. Audio recordings were transcribed and analysed thematically. Expert review of the coding frame and dual coding of transcripts maximised rigour.

**Results:**

Twenty-eight GPs (10 fully qualified, 18 trainees) participated in five focus groups. Four major themes emerged: (1) why education is needed, (2) perceived educational needs, (3) learning preferences, and (4) evaluation preferences. EoLC was perceived as emotionally and clinically challenging. Educational needs included: identifying patients for palliative care; responsibilities and teamwork; out-of-hours care; having difficult conversations; symptom management; non-malignant conditions; and paediatric palliative care. Participants preferred learning through experience, working alongside specialist palliative care staff, and discussion of real cases, to didactic methods and e-learning. 360° appraisals and behavioural assessment using videoing or simulated interactions were considered problematic. Self-assessment questionnaires and patient and family outcome measures were acceptable, if used and interpreted correctly.

**Conclusions:**

GPs require education and support in EoLC, particularly the management of complex clinical care and counselling. GPs value mentoring, peer-support, and experiential learning alongside EoLC specialists over formal training.

## Background

General Practitioners (GPs) and other community healthcare providers are vital to the delivery of end of life care (EoLC) internationally, assuming overall responsibility for direct patient care, providing generalist palliative care, ensuring co-ordination and communication with colleagues and social care providers, and preventing unnecessary hospital admissions [1–4]. In England, where the current study was conducted, GPs’ responsibilities now include commissioning of services in local communities. Working in Clinical Commissioning Groups (CCGs) with other practices, GPs are key players in making the wider systemic changes required to improve EoLC [5] and help shift care from hospitals to the community, in line with patient wishes [6, 7].

However, there is evidence that GPs encounter challenges in providing EoLC and that the quality of EoLC by GPs can be problematic [8–11]. A recent survey of bereaved relatives of 596 people who died of cancer in London, UK, found that GPs received significantly less favourable ratings than district, community or private nurses providing homecare and specialist palliative care providers, with 28.2% of GP care rated very poor, poor, or fair [12]. However, EoLC is a relatively small component of a GP’s workload and staying abreast of developments in policy and practice presents challenges [11, 13–15]. Furthermore, training in EoLC for general practice has its limitations [15–17]. A systematic review of palliative care delivery by GPs found that many GPs feel ill-prepared and lack confidence in EoLC, despite being considered vital players in its delivery, and despite valuing this part of their work [9]. GPs reported feeling particularly challenged in managing patients’ and family carers’ psychological needs, pain and other symptoms. Policy guidance is right to recognise the urgent need for evidence-based education in EoLC for GPs [5, 18].

The literature describes several GP training interventions. Yet their evidence base is often unclear and few have been rigorously evaluated [19]. Where training is based on evidence, it usually concerns the training and education GPs require [20], rather than their learning preferences [17, 21]. In the UK, a pilot study of training and support materials (leaflets, postcards and posters) for GPs was conducted in 2011 by the Dying Matters Coalition [22]. However, of the 59 participants, only 25 attended a training workshop, and it is unclear how the content and format of the training were developed. While the Gold Standards Framework (GSF) team offers GP training on the use of the GSF as a set of guidelines, mechanisms and assessment tools, up to now evaluation has focused on categorical questions about the implementation of organisational and clinical processes, and self-rated assessments of quality associated with palliative care provision, rather than staff, patient or family outcomes [23, 24]. No rigorous evaluations exist of GP EoLC training in the UK [18]. Outside the UK, evaluations of GP EoLC education have had mixed results, with a recent controlled trial of a palliative care training programme for GPs finding no significant effect on communication skills or patient outcomes [25, 26].

To inform the development and evaluation of UK EoLC education for GPs, we aimed to explore their educational needs, preferred learning methods, and acceptable methods of evaluation.

## Methods

### Study design

Face-to-face semi-structured focus groups were conducted with trainee and qualified GPs.

### Setting

Participants either worked at a CCG or were trainees completing their specialist training (ST1, ST2 and ST3 years; ST1 is the first year and ST3 the last) in a large UK city. Focus groups were held at convenient times and locations around existing events so as not to interfere with work or study schedules: one took place at a conference centre where an event on cancer care in primary care was being held, one at the research department in line with the schedules of interested participants, and three at a residential conference centre where an away day for trainee GPs was scheduled.

### Sampling and recruitment

Inclusion criteria were: practicing as a GP or training to be a GP. Practicing GPs were recruited in two ways: advertisement in the electronic bulletin to the CCG mailing list, and in-person by the researchers at the event on cancer in primary care. Trainees were recruited in-person by the researchers during the away days. Maximum variation sampling aimed to capture diversity of age, years of experience, gender, and ethnicity. Data collection continued until data saturation was reached [27].

### Data collection

Focus groups were facilitated by experienced researchers with academic backgrounds in palliative care and health psychology and an interest in the education of healthcare professions (LS or LB), following best practice [28]. Another researcher or research administrator (LB or LK) attended each focus group to take field notes on environmental factors and non-verbal behaviours during and immediately after each group. The researchers and administrator were not known by participants and introduced themselves as academic researchers unconnected to the CCG or their training programme. Guidelines including maintaining confidentiality, allowing all participants to speak and there being no right or wrong answers, were explained at the beginning of each focus group. The research was part of a wider study generating data to inform future training in palliative and EoLC and its evaluation. We defined EoLC broadly as care of patients with incurable, progressive and life-limiting disease, and palliative care as an holistic approach to EoLC in line with the World Health Organization definition [29]. The topic guide (Table 1) was based on gaps in the existing literature, and revised with input from the project team and lay advisory group (one patient with advanced disease and four family caregivers). To prompt discussion, during the focus groups participants were given handouts listing the topics covered in a current two-day training on palliative and EoLC held in a local hospital [30] (Table 2), and asked to reflect on which of these would be relevant or not for GPs. The focus groups were recorded, transcribed verbatim and anonymised. Participants also completed a brief demographics form.

**Table 1:** Topic guide

Training experience
What sort of training, if any, have you already received regarding how to communicate with and support people with serious, life-threatening illness, and their families?
*Prompts: This can include undergraduate or post-graduate training, short courses, professional development courses, etc.*
Training topics [participants shown list of EoLC topics (Table 2)]
Are there any topics that would be helpful in an EoLC training course for GPs? Why?
Are there any topics that would not be helpful or relevant in an EoLC training course for GPs? Why not?
Are there any topics we haven’t mentioned that you think should be included in an EoLC training course for GPs? [Explore justification for these additional items]
Preferred course format /time/ delivery
How long should a course be? *Prompts: Would you prefer shorter sessions over multiple days, or fewer longer sessions? Two days? One day?*
When should it be held? *Prompt: Are particular times of day best?*
Who should attend? *Prompt: Would you prefer a course attended by many different healthcare professionals or GPs only? What benefits are there to multi-professional learning? What drawbacks?*
Who should teach the course? *Prompt: hospital/community palliative care staff? Other generalist providers e.g. GPs?*
How should it be taught? *Prompt: in-person versus online, as lectures versus interactive skills training. What about a mixture of in-person and online resources?*
Where should it be held? *Prompt: at a local hospital? Local hospice? Non-medical location?*
Mentoring / ongoing supervision techniques
Do you think that ongoing mentoring or supervision would be useful or not useful alongside an end of life care training course?
*Prompts: If yes, what do you think would be the best way to provide this? What are your views of mentoring by an expert by experience, i.e. patient/family member? If you don’t think mentoring/supervision would be useful, why not?*
Testing training effectiveness
How would you feel about us assessing the effectiveness of the training course by…
videoing or audio-recording your encounters with real or actor patients or families?
using patient or family satisfaction measures?
using 360° appraisals from colleagues, managers, patients and family members?
using process outcomes, for example referral to palliative care or place of death?
*Prompts: Are any of these methods particularly preferable or not preferable? Why?*

**Table 2:** Topic list

Understanding patients'/families' priorities in EoLC
Understanding your role in EoLC
Using the Amber Care Bundle [34, 35]
Helping patients achieve their preferred place of care
Fast Track discharges [37]
Understanding and managing common symptoms in dying patients
Understanding spiritual and cultural aspects of dying
Having difficult conversations with patients and families
Understanding advance care planning and using Coordinate My Care [37]
Understanding grief and providing support for family experiencing bereavement

### Analysis

Transcripts and associated field notes were analysed within a minimal realist paradigm [31] using thematic analysis [32] to describe the data and explore emerging patterns. This process comprised five stages: (1) Familiarisation with the data and inductive coding of five transcripts to generate a draft coding frame (LB); (2) Refinement of the coding frame with input from academic and clinical members of the project team; (3) Application of the final coding frame to all data (LB); (4) Independent dual coding of a sample of two transcripts by academic and clinical team members to assess inter-rater reliability [33] and refine the coding frame (JK, VR); (5) Review of all coding and generation of a narrative summary, paying attention to deviant cases within each theme and differences between trainees and qualified GPs [34] (LS). Analysis was managed in QSR NVivo 10 [35].

## Results

Data saturation was reached after five focus groups, totalling 28 participants. Mean length of the groups was 55 mins (range 35-82 mins). Participants comprised 18 trainees (8 ST1s, 3 ST2s, 7 ST3s) and 10 GPs (median years in practice 109, range 3-45). The majority were women (79%), with a median age of 32 (range 27-63) (Table 3). Four themes related to the study aims: why education is needed, perceived educational needs, and preferences for learning and evaluation.

**Table 3:** Demographic characteristics

ID	Job Title	Years in practice/training	Gender	Age Group	Ethnicity
GP01	GP	15	F	40–49	White British
GP02	GP	5	M	30–39	White British
GP03	GP	45	M	60–69	White British
GP04	GP	20	F	40–49	Sri Lankan
GP05	GP	4	M	30–39	White and Black African
GP06	GP	6	F	[not disclosed]	Pakistani
GP07	GP	3	F	30–39	Black African
GP08	GP	12	F	40–49	White British
GP09	GP	35	M	60–69	White British
GP10	GP	3	F	40–49	Black African
GP11	GP Trainee	ST3	F	30–39	White British
GP12	GP Trainee	ST3	F	30–39	White British
GP13	GP Trainee	ST3	F	30–39	White British
GP14	GP Trainee	ST2	F	20–29	Indian
GP15	GP Trainee	ST1	F	20–29	White British
GP16	GP Trainee	ST2	F	20–29	Indian
GP17	GP Trainee	ST1	M	30–39	White British
GP18	GP Trainee	ST1	F	20–29	Pakistani
GP19	GP Trainee	ST1	F	[not disclosed]	Pakistani
GP20	GP Trainee	ST1	M	20–29	White British
GP21	GP Trainee	ST2	F	30–39	Other Mixed/multiple
GP22	GP Trainee	ST3	F	30–39	White British
GP23	GP Trainee	ST3	F	30–39	White British
GP24	GP Trainee	ST1	F	20–29	[not disclosed]
GP25	GP Trainee	ST3	F	30–39	White British
GP26	GP Trainee	ST3	F	30–39	White British
GP27	GP Trainee	ST1	F	30–39	White British
GP28	GP Trainee	ST1	F	20–29	Indian

## Why education is needed

All participants recognised the importance and relevance of EoLC: *‘Generalists should be specialists in end of life care because we are the coordinators at the end.’* (GP04). All except one participant believed there to be a need for more or better education in EoLC. The reasons given fell into four sub-themes: attrition of skills and the difficulty of keeping up to date; inadequate exposure to care of the dying during training and in clinical practice; a lack of confidence, and the complexity of EoLC. One GP felt that training was not the answer to improving EoLC: *‘The training model is one that I would question, because I don’t think we can train people to do these things. We can certainly help, help people develop. It's like training people in music or art or philosophy or something: it has to be inductive rather than structured… it's a matter of mentoring’* (GP03).

### Attrition of skills and difficulty of keeping up-to-date

Qualified GPs reported attrition in their symptom management skills, using drug charts and setting up syringe drivers: *‘Unless you are doing it and getting experience prescribing and seeing patients you forget it instantly’* (GP11). EoLC education received during GP specialisation, particularly regarding symptom management, was lost over time from lack of exposure and reliance on palliative care specialists or colleagues with a special interest in palliative care (e.g. Macmillan GP facilitators): *‘You're trained to quite a high level. The difficulty is in real life… it's hard to maintain that, I would say particularly if you've got people that are very, very skilled like [Hospice] who take on a big burden of the palliative care… You end up doing less, so you become less skilled, particularly with things like medication use’* (GP02).

### Inadequate exposure to care of the dying

Both established GPs and current trainees reported inadequate, inconsistent education in and exposure to EoLC during undergraduate training and GP specialisation. Palliative care education was described as ‘opportunistic’, dependent on where and with whom you worked: ‘*If you happen to be rota’d onto a care of the elderly job, better for you, because you get to learn about it, but if you are not…’* (GP14). This included ST3s soon to complete GP specialisation: *‘I feel like I’d need a lot of support, so [EoLC]’s something that I need to try and get involved in before my training ends in three months’ time. I don’t feel that I’d be competent’* (GP25). Amongst trainees, palliative care experience was more often gained in hospital placements than in the community (*‘In the GP placement, a lot of the palliative patients go to the partners who know the families’* (GP25)), but this was inadequate: *‘A hospital is completely different… you have the support of nursing staff and… a palliative care team at the hospital… In general practice you don’t have as much support’* (GP21).

### Lack of confidence

Due to limited palliative care education and experience, there was a widespread lack of confidence in EoLC amongst both qualified and trainee GPs. This was reported to lead to hospital admissions (‘*send them into a hospital and they can work out if it is reversible or not’* (GP08)) and poor symptom control: *‘I have bottled it more than once and under-treated somebody’s pain because I am scared of giving out that much morphine.’* (GP12). Confidence was lacking in particular topics: providing out-of-hours care, symptom management, identifying patients at the end of life, initiating palliative care, and some aspects of communication (e.g. initiating discussions about palliative care and communicating with families). Experience of managing EoLC in the community increased confidence: *‘[My GP trainer and I] had… a handover session with a patient and then, after that, I saw her on my own, with support from my trainer if I needed it, and that worked really well… it made me feel confident.’* (GP21), and this practical clinical experience was seen as invaluable: *‘Current registrars have a full curriculum statement [on EoLC] devised by locals GPs… But I think it is the practical skills that you need.’* (GP04).

### The complexity of end of life care

However, both experienced and trainee GPs reported EoLC to be complex and challenging in itself (*‘When you are just starting out, it is really scary’* (GP10). EoLC could be personally difficult (*‘a big emotional burden… exhausting.’* (GP02)), in part because *‘there isn’t an instruction manual and it depends on our humanity… and actually we all become helpless and powerless’* (GP03). The real-life complexities of providing EoLC were challenging: communicating well with patients when time is short, resistance among patients to discussing death or dying, using local systems, or justifying a decision not to refer a patient to hospital or to stop their medication: *‘When [the] specialist system doesn't understand what you are trying to do in primary care, you kind of start to fall apart already, because… someone along the line might challenge you and say, well, the microbiologist said you should do this and you haven't… So as much as you want to keep people at home, sometimes the specialist that you depend on… directs what you do.’* (GP07). Although GPs who trained recently reported having more theoretical training in EoLC than those trained many years previously, this training often failed to acknowledge the complexities of real-life practice; for example, the difficulty of enabling a home death in line with a patient’s wishes: *‘I have so many examples of palliative care patients who had all the conversations… They are on that register they are always trying to get us to put people on, and then they still end up going and dying in hospital… You have spent however many hours, and you think, what really have I achieved here?’* (GP05).

## Perceived educational needs

Education needs arose in nine sub-themes: identification and referral for palliative care; local services and resources; local systems and frameworks; roles, responsibilities and teamwork; out-of-hours care; difficult conversations and counselling skills; symptoms and medication; caring for patients with non-malignant conditions; and paediatric palliative care (Table 4). Awareness of local and national initiatives such as the Amber Care Bundle [36, 37], the Gold Standards Framework [24, 38], Coordinate My Care [39] (a regional, electronic multi-access clinical care record) and procedures for Fast Track discharge [40] and out-of-hours care was patchy. GP trainees felt that communication skills were well covered in current training (*‘I don’t think communication issues we need more of, I feel like we do those to death in GP [training], no pun intended there.* [Laughter]’ (GP11)), although some felt their skills could be better: *‘I feel from all the role play and stuff we have done we are quite good at bumbling our way through it competently, but [we] could be more effective.’* (GP12). While participants in general felt they had received enough communication skills training, initiating discussions of palliative care and provide emotional support to families nevertheless remained difficult.

**Table 4:** GPs’ perceived training needs

Training need	Exemplifying quotation
Identifying and referring patients for palliative care	*‘Identifying which patients need to be highlighted and prioritising those patients… I don't think we really do that very well. I think they slip through the net quite easily.’* (GP19) *‘Whether everyone needs to be referred to palliative care, or if there are people that we can manage in the community without input, if we feel comfortable enough – I wouldn't know that decision. I would always refer, because… I'm not comfortable with any of the medications.’* (GP30)
Local services and resources	*“Practical things - what do you actually do, who do you actually phone up, who’s involved in that team… there must be… all kinds of services going on that we just don’t know about… that would be useful, to see ‘these are all the people, these are all the resources you potentially use and this is how you get hold of them.’”* (GP02)
Local systems and frameworks	*‘How does this system work in my locality? How do I contact this person? What advice, specifically, do I give? What are the guidelines in my area? Where do I access them? That kind of thing.’* (GP29) *“In my practice, some people don't know about hospital at home. So I’ve sent an email round saying, do you know you can do this? It is really interesting. You will have five GPs and only one will know about hospital at home or only one will know about ‘Talk Kings’ where you can get 24 h access to a consultant. And it is like it is really bitty how we get our information.”* (GP09)
Roles, responsibilities and team work	*‘You’ve got your role in their care, but knowing what you can legitimately expect of other team members, and what it's not bad to ask them to do, or what they may offer in terms of support and things, I think that's quite important, because I don't feel like I've got a good grasp of exactly what everyone could do in that team.’* (GP19)
Out-of-hours care	*‘I have had some phone calls about palliative patients in out of hours that [are]… more challenging because you really just don’t have any, well, very little background.’* (GP25)
Difficult conversations and counselling skills	*‘I don't feel particularly skilled and I find it especially difficult if you've been with - if you've known a family for years… but then you realise that, Dad's actually… this is the beginning of his dying process… how do you have that first conversation?’* (GP02) *‘We’re all expected to be counsellors, although none of us have had any counselling training, so if there were some sort of people from the mental health team or counsellors that could come in and offer… sessions… that would be useful.’* (GP03)
Symptom management	*‘I know a drug chart, whenever I get one, I get really flustered because there are rules and criteria and different things.’* (GP09) *‘For me it is all the practical stuff that you guys mentioned, like drug doses and these whacking great doses of morphine and is it saline or is it water or whatever.’* (GP16)
Caring for patients with non-malignant conditions	*‘It is easier when they have got a terminal diagnosis like a cancer or something. When they have lots of comorbidities and they are just starting to fail, that is really hard.’* (GP13)
Paediatric palliative care	*‘Paediatric terminal care, it's something that, thankfully, we don't see very much of and in fact are very ill equipped to deal with I think… on the rare occasion that it does come, come along.’* (GP03)

## Learning preferences

Both qualified and trainee GPs considered real-life experience of EoLC the gold standard: *‘No more lectures. No. We need experience’* (GP07). Participants valued highly placements at hospices and opportunities to shadow, discuss cases with and share care with specialist palliative care staff: *‘What I am astounded by is actually the benefit and the close working when it goes well with our community nurses and with our specialist palliative care. It is brilliant. Getting out and find the consultant there on a joint visit, you learn so much.’* (GP08). Face-to-face mentorship was suggested as a way of providing knowledgeable advice and a source of peer support that was particularly needed in EoLC (*“[someone] to turn to, to sort of say, 'that was quite rough’”* (GP02)).

As time is at a premium, participants felt training in EoLC needed to be easily integrated into clinical practice, existing meetings and training events. Multidisciplinary teachers and co-learners were favoured. Learning preferences reflected the high value placed on real-life experience: participants were particularly keen to learn from palliative care specialists or experienced GP mentors, and felt that reflection on real case studies in training was more effective for learning than didactic methods (‘*give a real life situation – what would you do, I think – rather than the theory, just the theory.’* (GP23)). A role for services users was also suggested: *‘discussions with actual relatives who have gone through it… seeing what they felt was good, what they felt was bad.’* (GP24). E-learning elicited mixed views. Trainees were used to online learning and understood its value in terms of cost-effectiveness and flexibility, but several reported that they found online learning difficult to absorb and retain: *‘You feel like a real expert for the first six weeks after you do it and then you forget it.’* (GP11)*.* Done well, role plays could be effective and memorable; however, trainees seemed saturated with the technique by their third year: *‘There is only so much you can get out of pretending to tell your friend they are dying and having somebody observe you do it. I think I stopped learning from that about a year ago.’* (GP12).

## Evaluation preferences

Participants questioned the accuracy of 360° appraisals in assessing quality of care: ‘*You pick the people… and they’re probably people that you’ve had more interaction with’.* (GP25). Participants felt that behavioural assessment using videoing was not acceptable unless it was required for a specific qualification. Several participants were averse to any form of behavioural evaluation: *‘I personally avoid anything that’s like a role-play where you’re assessed. So, if there’s like a simulation day, where I know you’re going to be watched and assessed, I won’t apply for it.’* (GP24). Trainees also questioned the validity of simulated behavioural assessments as a way of evaluating communication skills, due to the ability to learn exam technique: GP 16: *‘[Simulations] probably wouldn’t give you an accurate reflection of the course.’* GP13: *‘Actually, if we are getting into role play, you can learn how to tick the boxes quite easily.’* [General sounds of agreement] GP13: *‘We are masters at role play now.’*

Self-assessment questionnaires to test the effects of education on confidence and knowledge were considered of limited use, but respondents reported answering honestly and felt they were acceptable if not too lengthy or burdensome: *“We're notoriously bad at knowing how good or bad we're probably doing in a consultation. So… saying, ‘Yes, I feel more confident now,’ or, ‘I think that I'm better now,’ I don't think we're the best judges of the actual ultimate outcome”* (GP17). Most GPs were in favour of patient and family feedback if done sensitively, (‘*asking them about their experience, what could be done better, what do they feel was missing’* (GP24)) but some felt this inappropriate: *“Saying to a relative or a patient, ‘How do you think your doctor did in treating you as you’re dying?’ is the last thing they want to be thinking about, this filling in a questionnaire.”* (GP20). Timing patient and family outcome measurement sensitively was considered important, as was the way in which data are collected: *‘If it’s a familiar face doing that that would be okay, but I think if it came in the post it could be quite upsetting, or probably the return rate would be quite low.’* (GP02). GPs were aware of possible confounders in measuring the effects of education on patient and family outcomes, e.g. *‘People who return those [questionnaires] are going to be either really hacked off with you or really impressed.’* (GP16).

## Discussion

This study is one of the first to examine the needs and preferences of GPs and GP trainees regarding education in EoLC and how its effectiveness might be evaluated. Previous studies in the UK have explored education needs amongst GPs conducting out-of-hours care [17], but not amongst GPs with different levels of training and experience. This study identifies unmet educational needs in EoLC: participants reported that skills erode and remaining up to date is difficult, exposure during training is inadequate and inconsistent, and in clinical practice palliative care is complex and their confidence low. A key finding is that qualified GPs and trainee GPs valued mentoring, peer-support, and experiential learning alongside specialists in EoLC, over attending formal training. In terms of formal education, real case studies were valued more highly than didactic methods, and e-learning EoLC was not considered appropriate. Respondents emphasised that any formal education needed to be easily integrated into clinical practice and existing meetings. In terms of evaluation, participants felt 360° appraisals were inaccurate, and questioned both the validity of assessments using simulated interactions and the acceptability of behavioural assessment using video. Self-assessment questionnaires and patient and family outcome measures were largely considered acceptable and potentially useful, if used and interpreted correctly.

Our findings support other studies in highlighting the limitations of current end of life care education for GPs and their lack of confidence in this area [9, 15–17], as well as educational needs relating to out-of-hours care [17], symptom control [20, 41, 42], non-cancer diagnoses [17, 20, 43], and counselling [16, 20]. Where we add to the evidence is in identifying the need for practice-based mentorship and/or apprenticeship models in education in end of life care. We also describe specific educational needs around the identification and referral of patients for palliative care; roles, responsibilities and team-working; local services and systems; and in paediatric palliative care. Of note, participants in general felt they had had sufficient communication skills training, despite still finding conversations about dying to be difficult. Perhaps what is needed is not more communication skills training, but qualitatively different communication skills training.

As in a study by Pype et al. in Belgium [21], we found that practical experience, including workplace learning through collaboration with palliative care specialists, was highly valued as a way to learn skills. Our participants were unanimously positive about collaborating with and learning from palliative care specialists. This suggests improvements in this area since 1998, when a study found that GPs questioned the extent to which they should defer to the specialist palliative care team [43]. Our finding that GPs had reservations about learning EoLC in an online format, preferring face-to-face education, contradicts other studies. A survey of GPs (*n =* 203, 20.3% response rate) working for an independent provider of out-of-hours services in England found that e-learning was the preferred method (67.5%, *n =* 137) [17]. A randomised controlled trial in Spain [44, 45] also found that primary care physicians were satisfied with an online palliative care education programme. However, that programme integrated mentoring, which GPs in the current study also valued. E-learning in this area warrants further research.

A strength of this study is the diverse sample: clinicians ranging from junior trainees to experienced GPs, good representation from both genders, and participants of diverse ethnicities. However, as participants were recruited in a major city our findings might not be directly transferable to non-urban areas or other countries, where access to education and educational needs will differ [20]. GPs who participated in this study may also have had a particular interest in EoLC, and GPs with less interest may report different needs and experiences. In particular, GPs who did not attend due to lack of interest or time constraints may have even greater unmet and unknown needs. Finally, although we identified and discussed differences in the views of experienced GPs and GP trainees, the study was not designed to compare these groups in detail; this would be an interesting area for future research.

The clinical implications of the study include an identified need to improve GP education over several domains, primarily through implementing service models and interventions that provide access to experiential learning, mentorship and joint working with specialist palliative care providers. This may require a paradigm shift away from thinking of formal training as the best way to educate healthcare professionals, particularly in EoLC, which can be both personally difficult and clinically complex. Our findings suggest that formal education which is not supported and enhanced by real-life experience, resources and mentoring may be insufficient to bring about sustainable improvements in EoLC in primary care and a potential waste of resource. While this study was conducted in the UK, we suspect the need for experiential learning and mentorship in end of life care education is more widely applicable internationally. For example, we believe the standard education for physicians in the United States, the Education in Palliative and End-of-Life Care programme, does not currently use mentored real-life clinical situations to teach physicians in practice and in training how to provide EoLC. Further research is needed to adapt and evaluate service models and interventions to improve EoLC in primary care in local settings [46, 47] to identify best practice in GPs’ education and support. In the UK, Macmillan GP facilitators funded since 2011 could play an important role in supporting primary care staff and enhancing education.

With the caveat that formal education must be implemented alongside systemic changes, this study also provides guidance for the formal education of GPs in EoLC. In the UK, the RCGP Palliative and End of Life Care Toolkit [48] to support GPs’ provision of EoLC could be enhanced to include a more comprehensive, searchable database of training available to GPs and associated evidence. A current interdisciplinary course (Transforming EoLC) that is run for a variety of EoLC providers [30] incorporates some of the topics identified here. The course is highly evaluated and further research into its effectiveness warranted [30]. However, four topics are not covered in the generic course: identifying and referring patients for palliative care; out-of-hours care; caring for patients with non-malignant conditions; and paediatric palliative care. These topics may be of specific relevance in developing stand-alone education on EoLC for GPs. It is essential that any education rolled out for GPs is evidence-based and evaluated for effectiveness.

## Conclusions

GPs require education and support in EoLC, particularly the management of complex clinical care and counselling. GPs value mentoring, peer-support, and experiential learning alongside EoLC specialists over formal training. Findings from this study can help inform EoLC educational and support interventions for GPs and guide the evaluation of such interventions.

## Abbreviations

CCGs: Clinical Commissioning Groups
EoLC: End of Life Care
GP: General Practitioner
GSF: Gold Standards Framework
ST: Specialist Training
